# Omicron variants of SARS-CoV-2 and long COVID

**DOI:** 10.3389/fimmu.2022.1061686

**Published:** 2022-12-09

**Authors:** Chengliang Yang, Hedi Zhao, Casey P. Shannon, Scott J. Tebbutt

**Affiliations:** ^1^ Prevention of Organ Failure (PROOF) Centre of Excellence, St. Paul’s Hospital, University of British Columbia, Vancouver, BC, Canada; ^2^ Centre for Heart Lung Innovation, Providence Research, St. Paul’s Hospital, Vancouver, BC, Canada; ^3^ Division of Respiratory Medicine, Department of Medicine, University of British Columbia, Vancouver, BC, Canada; ^4^ Department of Surgery, University of British Columbia, Vancouver, BC, Canada

**Keywords:** SARS-CoV-2, omicron variant, COVID-19, long covid, post COVID-19 condition, vaccine

## Abstract

Understanding the epidemiology of long COVID and emerging variants has significant public-health implications as physical interventions and restrictions that help limit viral spread are eased globally. Here, we provide rationales for the necessity of updating current vaccines to improve protection against omicron and emerging variants, as well as more research into understanding the epidemiology and mechanisms of long COVID.

## Long COVID overview

Long COVID (or post COVID-19 condition) describes a range of symptoms such as chest pain or tightness, cough, fatigue or breathlessness, ageusia and anosmia, headache, insomnia, anxiety, and depression, which can last for months after varying severities of SARS-CoV-2 infection (1–4). Many patients with long COVID have difficulty returning to baseline functional capacity and have increased healthcare service utilization rates. Long COVID has significant negative implications for patients, caregivers, health systems and society (5). A conservative estimate suggests that more than 200 million people globally suffer from long-term effects of COVID-19 (6), although the prevalence of long COVID estimates can vary widely given the incongruity in case definition (e.g.World Health Organization (WHO), the United Kingdom National Institute for Clinical Excellence (NICE) and the United States Centers for Disease Control and Prevention (CDC)). The statistics are worrisome, and little is known about what triggers long COVID, how to prevent it, and how to treat it. Long COVID has seriously challenged the outlook of current therapeutics and SARS-CoV-2 vaccines.

## What are the epidemiological features of the SARS-CoV-2 Omicron variant?

**Figure 1:**
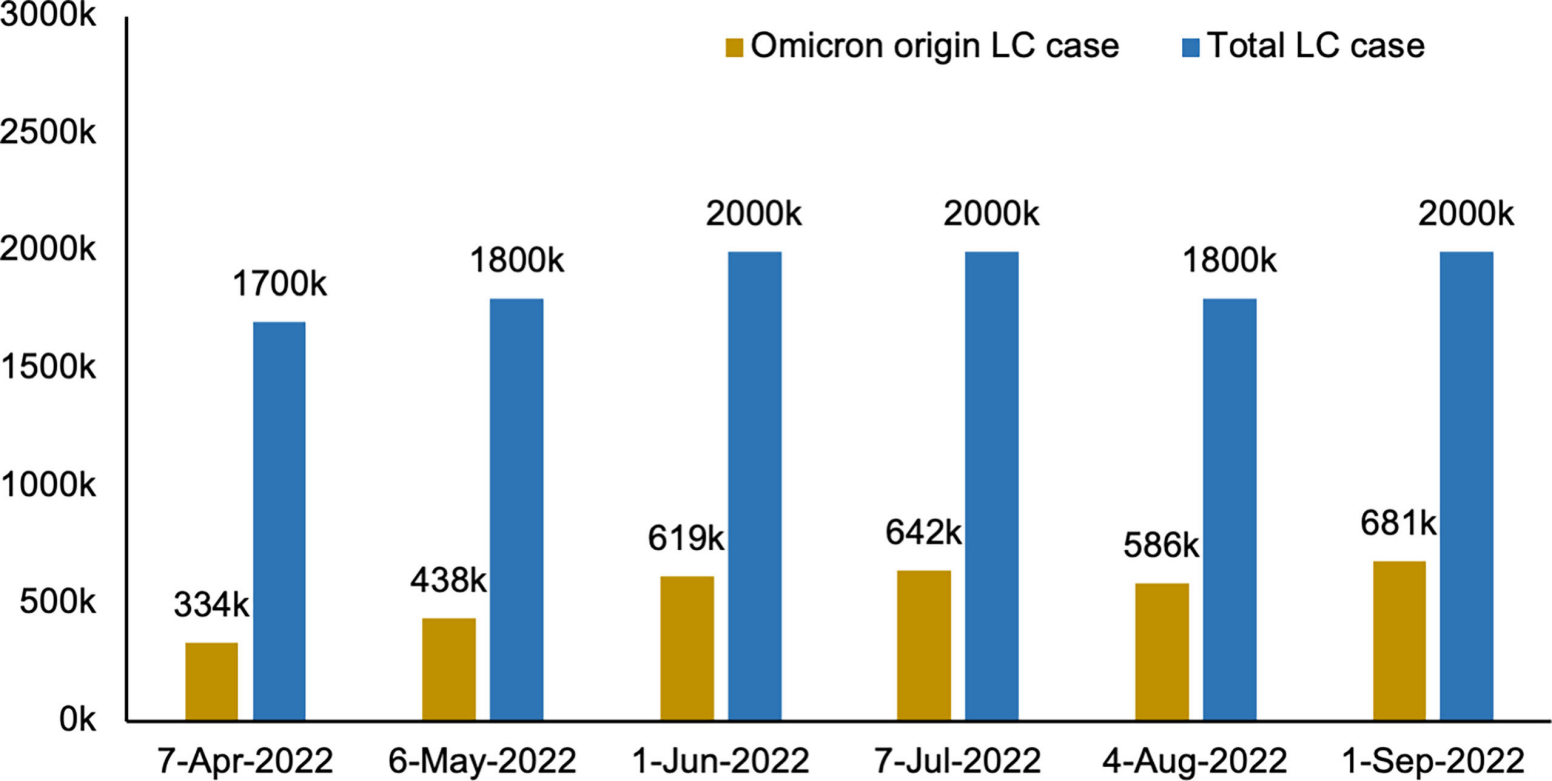
Estimated number of people living in the U.K. with long COVID of Omicron origin. LC, long COVID; K, thousand. “Data source: Office for National Statistics. https://www.ons.gov.uk/peoplepopulationandcommunity/healthandsocialcare/conditionsanddiseases/bulletins/prevalenceofongoingsymptomsfollowingcoronaviruscovid19infectionintheuk/previousReleases”.

The most prevalent Omicron variant, B.1.1.529, contributes to approximately 34% (681,000) of the U.K.’s reported 2.0 million (3.1% of the U.K. population) self-reported long COVID cases according to the most recent Office of National Statistics (NOS) report on the national prevalence of long COVID in the U.K. (September 1^st^, 2022; **Figure 1**) (7). Omicron is highly variable: there are multiple and increasing PANGO (Phylogenetic Assignment of Named Global Outbreak) sub-lineages associated with the Omicron variant of concern (VOCs) and the main BA.1, BA.2, BA.3, BA.4, BA.5 and descendent lineages may also have their own sub-lineages. Importantly, the risk of reinfection is higher with the omicron variant compared to the beta (B.1.351 and descendent lineages) and delta (B.1.617.2 and descendent lineages) variants (8, 9), raising concerns about immune escape.

## What are the underlying pathophysiological mechanisms of long COVID?

Combined, SARS-CoV-2 and its variants have infected more than 611 million people worldwide and killed more than 6.5 million (as of September 16, 2022; https://coronavirus.jhu.edu). The rapid surge in omicron variant infections poses a significant public health crisis with an unclear timeline for its progression as it already surpassed optimistic estimates for its end in March 2022 (10). Many will go on to develop long COVID, thus understanding the disease mechanisms will prove crucial in treating these patients. Phetsouphanh et al. analyzed blood samples from people with long COVID and found that immunological dysfunction with lymphopenia and increased expression of inflammatory mediators in these long COVID patients persisted for 8 months after the initial mild to moderate SARS-CoV-2 infection. This study suggested that the inflammatory mediators interferon beta (IFN-β), pentraxin 3 (PTX3), interferon gamma (IFN-γ), lambda interferon (IFN-λ2/3) and interleukin 6 (IL-6) were highly associated with long COVID (11). Still, whether these biomarkers can provide insight into the risk of developing long COVID secondary to omicron remains unclear. In a recent study, Naranbhai and colleagues showed that spike mutations in Omicron could escape T-cell responses in an HLA-dependent manner (12). Notably, a research group at the Oxford University recently reported an association of HLA alleles, specifically the HLA-DQB1*06 alleles with higher antibody response following immunization against the SARS-COV-2 vaccine and with a lower risk of breakthrough SARS-COV-2 infection (13).

## Do COVID-19 vaccines reduce the risk of long COVID after omicron infection?

From a public health planning perspective, it will be critical to understanding the effectiveness of COVID-19 vaccines for clinically meaningful outcomes such as hospitalization, admission to the intensive care unit, and death in the omicron era. There is a clear and urgent need for treatment options for SARS-CoV-2 variants such as delta- or omicron-induced long COVID. In The Lancet, Antonelli and colleagues report a lower risk of long COVID in vaccinated individuals with omicron (BA.1 period) compared to delta variant (14). Importantly though, their study did not consider reinfections. People with breakthrough infection exhibited a higher risk of death and a higher incidence of post-acute sequelae of COVID-19 compared to those without breakthrough infection in a study of more than 13 million people (15). Moreover, vaccination against SARS-CoV-2 offered less protection against lingering symptoms than expected (reducing the relative risk of long COVID by only about 15%) (15). Kared et al. demonstrated that individuals with Omicron SARS-CoV-2 breakthrough infections had activated SARS-CoV-2 wild-type Spike-specific cytotoxic T cells, activated follicular helper (TFH) cells, functional T cell responses, enhanced humoral responses, and rapid release of Spike and RBD-specific IgG+ B cell plasmablasts and memory B cells were significantly enhanced through rapid release into the circulation (16). Although strains may share a common ancestor and sub-lineage, there may be significant point mutations and antigenic changes between evolving strains of the same sub-lineage (e.g., BA.2.12 versus BA.2.12.1), resulting in different antibody cross-neutralization upon infection (12). As a result, reinfections and breakthrough infections may result in a resurgence of long COVID. Given the availability of data on breakthrough infection cases in many study groups, future studies may benefit from quantifying and elaborating on these patients’ demographic and health characteristics and delineating differences between delta and omicron variant breakthrough cases. Determining breakthrough infection rates following COVID-19 vaccination can be challenging and requires well-designed population-based cohort studies on vaccine efficacy and the risk of developing long COVID after Omicron infection. Such information could improve our understanding of how vaccine-induced immunity protects against breakthrough COVID-19-infection.

There is growing evidence that newer omicron variants have evolved to evade pre-existing antibody responses. For example, omicron BA.4 and BA.5 subvariants appear to be growing in prevalence and may have the ability to evade antibodies from earlier Omicron infections (e.g., BA.1) and/or BA.1-derived vaccines more effectively (17). A proportionately greater number of cases with BA.2.2 breakthrough infection had received three vaccinations compared to those with BA.1 breakthrough infection (18). In contrast, BA.4 and BA.5 have the strongest neutralization escape advantage in the unvaccinated population (19). In vaccinated individuals infected with BA.1, there was a 3.2-fold decrease in the neutralization level of BA.4 and a 2.6-fold decrease in BA.513 compared to BA.1.The emerging SARS-CoV-2 subvariants such as BA.2.12.1, BA.4 and BA.5 may cause a new wave of COVID-19 this winter. Although the Omicron booster shots (containing two mRNAs encoding the ancestral SARS-CoV-2 and the prefusion-stabilized spike glycoproteins of the omicron variant [BA.1]) are now available, the effectiveness of the bivalent omicron-containing mRNA-1273.214 booster vaccine remains unclear (20). A recent systematic review study suggests that the effects of COVID-19 vaccination on the risk of individuals with long COVID are still controversial, with some studies showing changes in long COVID symptoms after vaccination and others not (21). As vaccination programs continue, public health researchers must better understand how vaccines and Omicron affect long COVID rates and severity, with the long-term consequences of COVID-19 being considered by policymakers and supporting the need for next-generation COVID-19 booster to delivery strong immune response against VOCs, caused by omicron or other emerging variants.

## The psychological impact of long COVID on families and caregivers of patients

The COVID-19 pandemic has significantly disrupted COVID-19 patients’ and frontline healthcare workers’ mental health in many countries worldwide while the demand for mental health is increasing (22–24). Notably, the impact of long COVID on the mental health of patients’ families and caregivers of patients has not been well studied to date. However, there is evidence that most caregivers of critically ill patients experience high levels of persistent depressive symptoms and may experience physical and psychological morbidity (25–27). Governments will need to redirect their focus to health systems management and supportive policies to assess the initial impact of long-term COVID at the community or population level on families’ general mental health and psychosocial morbidity, and to provide early data to inform relevant policy and program actions.

## The current state of vaccinations

Globally, as of 16 September 2022, there have been 4,908,532,010 persons fully vaccinated (61. 55%), of which only 2,211,222,637 persons received booster vaccines (27.72%), reported to WHO (28). It remains of major concern that many health workers and vulnerable populations remain unvaccinated or are hesitant to be vaccinated (29). According to the 2022 WHO Strategic Preparedness and Response Plan, only 30% of healthcare workers and 20% of people aged 60 or older have been vaccinated in low-income countries (30).

## Summary and recommendations

According to the latest prospective cohort study, although booster vaccination resulted in a significant increase in anti-spike IgG responses, booster vaccination did not further enhance durable spike-specific T-cell responses following different COVID-19 vaccination regimens (31). Interestedly, the structural studies have shown that a receptor binding domain –specific human monoclonal antibodies 002-S21F2 potently neutralizes Omicron sublineages BA.1, BA.2, BA.2.12.1, BA.4, and BA.5 and previous VOCs without sacrificing potency (32). The development of a broadly neutralizing epitope for 002-S21F2 leading to a broad range of COVID-19 therapeutic and upgraded vaccines against most SARS-CoV-2 variants, including the Omicron sublineages, may be one of the effective strategies to combat pandemics in the future. If we were to propose that Omicron contributes to long COVID at the same rate as earlier variants, we could see a significant global health disaster next year. The global healthcare system will have to cope with more than 200 million patients with long COVID. Are we ready? Closing the COVID-19 vaccine equity gap is the best way to improve population immunity and protect against the next wave of outbreaks. Also, understanding the prevalence of long COVID and omicron variants among vaccinated and unvaccinated people, in both resource-poor and resource-rich societies, has urgent public-health implications as restrictions that limit viral spread are eased around the world. As vaccination programs continue, it is important for public health researchers to better understand how the vaccine and Omicron affect the long COVID rates and severity, place more emphasis on the long-term consequences of COVID-19, and support the need for new vaccines to provide better protection against Omicron and other emerging variants.

## Author contributions

CY, HZ, and ST conceived the original draft manuscript. CY, HZ, CS, and ST conducted the search and edit the manuscript. ST supervised this manuscript.CY, HZ, CS, and ST contributed to the manuscript and accepted the final version for publication.
